# Achilles' spear: A new therapeutic target for age‐related diseases

**DOI:** 10.1111/acel.14257

**Published:** 2024-06-21

**Authors:** Yang Xuan, Yue Duan

**Affiliations:** ^1^ The Second School of Clinical Medicine Zhejiang Chinese Medical University Hangzhou China; ^2^ Department of Urology The Second Affiliated Hospital of Zhejiang Chinese Medical University Hangzhou China; ^3^ Zhejiang Provincial Key Laboratory of Traditional Chinese Medicine Hangzhou China; ^4^ Zhejiang Provincial Key Laboratory of Sexual Function of Integrated Traditional Chinese and Western Medicine Hangzhou China

**Keywords:** age‐related diseases, miMOMP, mtDNA, SASP

## Abstract

The role of the senescence‐associated secretory phenotype (SASP) in the development of age‐related diseases is significant, and its control promises to have a tremendous positive impact on health. A recent study has identified a new mechanism for SASP regulation, titled miMOMP. Failure to regulate SASP would dramatically increase the risk of various age‐related health problems. Nonetheless, we have not completely comprehended how to modulate SASP. In this commentary, we summarise the specific mechanisms by which miMOMP regulates SASP and outline possible future research directions. Moreover, potential risks and obstacles to the clinical translation of miMOMP are also presented.

## Graphical abstract

Following anti‐miMOMP treatment, patients with age‐related diseases exhibited improvements. However, when these patients were treated with anti‐tumour therapy concurrently, the anti‐tumour effect diminished and tumour progression ensued. The conjecture about anti‐miMOMP treatment proposed in this paper may be an important research direction in this field in the future.

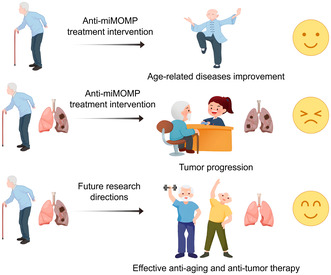

**Abbreviations**
APAF1: Apoptotic protease activating factor‐1
ISG: Interferon‐stimulated gene
MEF: Mouse embryonic fibroblasts
miMOMP: Minority mitochondrial outer membrane permeabilisation
MOMP: Mitochondrial outer membrane permeabilisation
mtDNA: Mitochondrial DNA
SASP: Senescence‐associated secretory phenotype
TFAM: Mitochondria‐derived transcription factor A

As people age, bodily functions decline, and cellular senescence begins to occur in tissues and organs, including the brain, bones and muscles, leaving them vulnerable to age‐related health problems, such as degenerative changes in organs and inflammation. During cellular senescence, there is a marked activation of SASP (Gorgoulis et al., 2019) components. Thus, controlling the induction of SASP may have profound implications for maintaining organismal homeostasis and managing disease. Apoptosis is a cellular fate quite distinct from cellular senescence, in which extensive mitochondrial outer membrane permeabilisation (MOMP) leads to cell death (Bock & Tait, 2020). However, MOMP occurring in a subpopulation of mitochondria is a feature of cellular senescence, at this point in time quite different from the situation in apoptosis again. Recently, Stella Victorelli et al. identified a mechanism known as minority MOMP (miMOMP) whereby the DNA nucleoids accumulate in the cytoplasm (Victorelli et al., 2023). This process involves the BAX and BAK macropores of the outer mitochondrial membrane, which allow the release of mitochondrial DNA (mtDNA) into the cytoplasm. Preliminary results indicate that removal of mitochondria in senescent cells hinders SASP and that during senescence miMOMP releases mtDNA into the cytoplasmic, triggering cGAS‐STING‐SASP pathway. The cGAS‐STING signalling pathway is a pivotal driver of chronic inflammation and functional decline during the ageing process (Gulen et al., 2023). Inhibiting this pathway may result in the reduction of inflammatory responses in aged cells and tissues, as well as the improvement of multiple peripheral organs and the brain. In the brain, the activation of the STING pathway leads to a transcriptional status in reactive microglia, neurodegeneration and cognitive impairment. The release of cytoplasmic DNA and the activity of cGAS play a significant role in aged microglia. Nevertheless, it remains uncertain whether the cGAS‐STING pathway is directly involved in cellular senescence or age‐related inflammation and dysfunction in human tissues. Inhibition of miMOMP in vivo led to a reduction in inflammatory markers and an improvement in several health parameters in aged mice. In addition, miMOMP showed a positive correlation with the activation of BAX/BAK.

Stella Victorelli et al. analysed proliferating and senescent human fibroblasts in order to investigate whether miMOMP is a feature of cellular senescence and verified that miMOMP occurs during cellular senescence. It was also investigated whether miMOMP is accompanied by BAX oligomerisation (i.e. BAX activation), and the experimental results showed a significant increase in BAX activation in senescent cells. This suggests that miMOMP occurs during senescence as a result of sublethal apoptotic stress.

It was demonstrated that the majority of cytoplasmic DNA in senescent cells mainly originated from mitochondria and that mitochondria‐derived transcription factor A (TFAM) was abundantly present in the mtDNA nucleoids in the cytoplasm of senescent cells, leading to the enrichment of TFAM in the cytoplasm of senescent cells, which in turn, is a preferred substrate for cGAS in conjunction with the mtDNA nucleoids conjugate.

Since miMOMP occurs only in mitochondrial subpopulations, Stella Victorelli et al. hypothesised that miMOMP is sufficient to drive senescence and SASP without causing apoptosis. The experimental results suggest that treatment of proliferating human fibroblasts with low concentrations of an activator of apoptosis revealed a significant increase in the downstream inflammatory factor component of SASP without affecting the rate of cell division, and an earlier arrival at the stage of replicative senescence in comparison to controls. This is also consistent with the results of their previous experiments. Given the observation that senescent cells show an activation of BAX, they then used CRISPR‐Cas9 gene editing to generate human fibroblasts defective in both BAX and BAK, verifying whether depletion of both BAX and BAK could inhibit the mtDNA release in the cytoplasm. Sure enough, the combined deletion of BAX/BAK inhibited DNA damage‐induced mtDNA release in senescent cells. And BAX/BAK regulated SASP but did not cause senescence‐associated cell cycle block.

Normally, MOMP leads to the release of mtDNA into the cytoplasm during apoptosis, but the inflammatory repercussions of this process are suppressed by caspases. Stella Victorelli et al. explored whether caspases also hinder miMOMP in senescent cells. Several experiments illustrated that caspases limit downstream inflammation driven by mtDNA in apoptosis. Nevertheless, caspases dependent on miMOMP do not impede SASP activation in cellular senescence (Figure 1).

**FIGURE 1:**
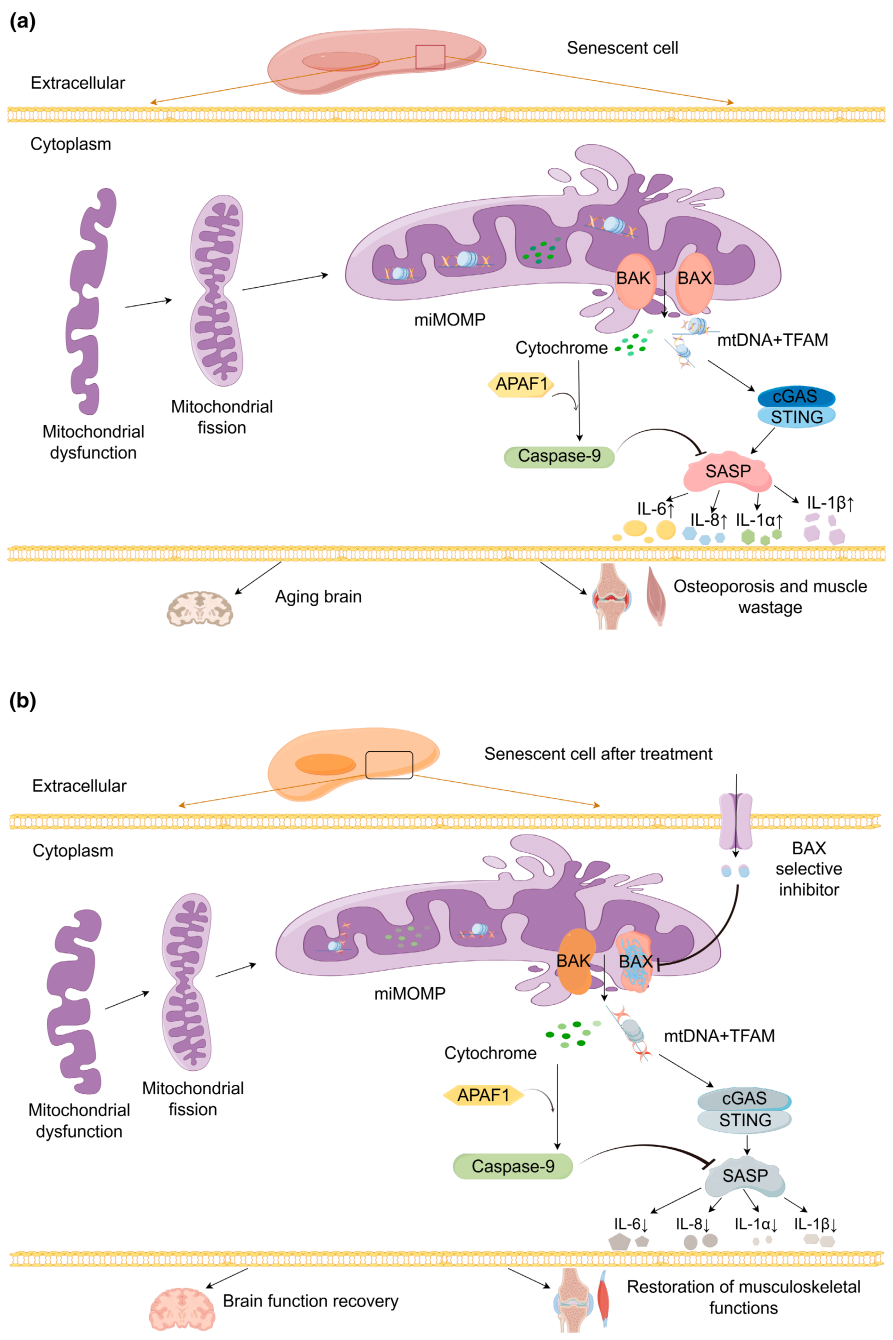
Schematic diagram of the mechanism of cellular senescence. (a) The key to the occurrence of cellular senescence is the SASP caused by miMOMP. Under normal circumstances, cytochrome C hinders SASP through APAF1‐dependent caspases. In senescent cells, a decrease in mitochondrial function results in mitochondrial harm and leads to BAK/BAX‐dependent miMOMP. mtDNA binds to TFAM, amasses in the cytoplasm and sparks the downstream cGAS‐STING signalling pathway, causing a significant upregulation of SASP‐related components. The functions of the brain, bones, muscles and other tissues and organs decline, thus augmenting the risks of age‐related diseases. (b) When senescent cells are pharmacologically intervened, the crucial BAK/BAX of miMOMP will be specifically targeted and inhibited, resulting in suppressed mtDNA accumulation in the cytoplasm. The accumulation of mtDNA within the cytoplasm will be inhibited, subsequently decreasing the expression of SASP components that follow. Finally, the restoration of brain and musculoskeletal function, among other areas, has been observed.

We already know that mtDNA can activate the cGAS‐STING signalling pathway and regulate SASP during apoptosis and after other stresses. Further experiments were conducted by Stella Victorelli and colleagues with the objective of elucidating the relationship between mtDNA, the downstream pathway of cGAS‐STING and SASP. They induced senescence in human fibroblasts and triggered extensive mitochondrial exhaustion. Finally, cells were transfected with mtDNA to measure their response. It was demonstrated that Parkin‐mediated mitochondrial clearance suppressed the expression of commonly known SASP components and that these components were subsequently upregulated upon transfection with mtDNA. mtDNA abnormalities resulting from TFAM deletion increased the expression of the interferon‐stimulated gene (ISG). It was found that most of the mtDNA stress‐induced ISGs were upregulated during senescence, decreased after mitochondrial clearance and recovered after reintroduction of mtDNA. Suggesting that TFAM deficiency in T cells accelerates cellular ageing, inflammation and senescence in mice. Using a more comprehensive database of NF‐κB pathway‐regulated genes and ISGs, they found a similar pattern. Furthermore, after experiments using wild‐type and TFAM^+/−^ mouse embryonic fibroblasts (MEFs, cytoplasmic mtDNA‐rich) cells, it was found that MEFs reach replicative senescence earlier.

Stella Victorelli et al. went on to find that the STING inhibitor did not prevent senescence‐associated growth arrest in MEFs or the expression of senescence‐associated markers; however, it blocked the expression of pro‐inflammatory factors. The CRISPR‐Cas9‐mediated deletion of cGAS and STING significantly reduced the secretion of SASP‐associated proteins and inflammatory factors. Although the secretion of SASP‐associated proteins and inflammatory factors resumed upon mtDNA manual transfection in cells, it was notably lower in STING‐deficient cells. This provides further backing to the hypothesis that mtDNA controls SASP via the cGAS and STING paths. Their experimental findings also show that mitochondrial hyperfusion averts the miMOMP and mtDNA release, and the mitochondrial dynamic driven MOMP in senescent cells is restricted to a subpopulation of mitochondria and regulates miMOMP‐induced SASP.

Finally, Stella Victorelli et al. evaluated the inhibition of mtDNA release and SASP by MOMP inhibitors. The results showed that BAX inhibitors performed as previously predicted, inhibiting the expression of SASP‐related factors in senescent cells. The ensuing animal and cellular experiments demonstrated that BAX inhibitor treatment ameliorated ageing‐related diseases, improved spine, femoral trabecular microarchitecture and skeletal muscle phenotypes, delayed disease progression and effectively reduced whole‐brain inflammation and expression of ageing‐related factors in aged mice.

In conclusion, Stella Victorelli et al. demonstrated experimentally that the release of DNA nucleoid bound to TFAM via mitochondrial subpopulations of BAX and BAK promotes SASP. During apoptosis, mtDNA is discharged into the cytoplasm via MOMP and activates the cGAS‐STING pathway, leading to SASP, but the presence of caspases inhibits its immune effects; in contrast, APAF1‐dependent miMOMP does not lead to apoptosis, and inhibition of caspases is not sufficient to inhibit cGAS‐STING signalling to the point of silencing its activation of SASP. It is interesting that the entry of herring testicular DNA into the cytoplasm to activate SASP does not depend on BAX and BAK. This observation has prompted us to hypothesise that mtDNA may have an independent mechanism to enter the cytoplasm and accumulate in testicular tissue, thus leading to cell senescence.

It is well known that MOMP is necessary for apoptosis, but miMOMP can cause cellular senescence without triggering apoptosis. Stella Victorelli et al. pioneered a novel mechanism by which mtDNA enters the cytoplasm to cause SASP in the hope of targeting the weakness of cellular senescence by inhibiting miMOMP, which could provide a new avenue for the treatment of delayed senescence and age‐related diseases. This could be a completely new therapeutic target. Thus for age‐related diseases, we could also envisage targeting mtDNA present in the cytoplasm of senescent cells that have not undergone apoptosis and pharmacologically inhibiting its downstream cGAS‐STING pathway to reduce SASP component expression; targeting a subpopulation of mitochondria in cells that have not yet senesced, and inhibiting BAX/BAK activity to block mtDNA release into the cytoplasm, thereby reducing SASP activation to improve health parameters. In addition, the present study deepened the link between mitochondrial dynamics and cellular end‐fate; previous studies have demonstrated that MOMP plays an important role in apoptosis; however, miMOMP in mitochondrial subpopulations promotes cellular senescence without apoptosis subverting the conventional knowledge. Therefore, further studies are necessary to investigate which part of the complete miMOMP‐promoted SASP pathway can be inhibited most efficiently to delay senescence, and to experiment multiple inhibitors in depth to determine the optimal efficacy.

Notably, on the other hand, what do we really know about the involvement of miMOMP in tumour immune mechanisms? It has been reported that limited MOMP (miMOMP) and subapoptotic (sublethal) activation of apoptotic factors play a key role in mechanisms such as transformation and immune escape of tumour cells (Han et al., 2020). During non‐apoptotic cell death, we have recognised how miMOMP, downstream activation of cGAS, STING‐IFN and other pathways, inducing sustained DNA damage, affects tumour cell biological changes. When cytoplasmic DNA is increased, this is more likely to activate the cGAS‐STING pathway when cells are exposed to external insults such as radiotherapy, which has been clearly demonstrated to play an active and important role in anti‐tumour immunity (Bao et al., 2020). Innate perceptual dependence on cytoplasmic accumulation of mtDNA is key to limiting tumour progression. Evasion of apoptosis is one of the main mechanisms of tumour resistance to current therapies. Therefore, many current anti‐tumour therapies focus on triggering more apoptotic tumour cells to reduce the tumour burden. Tumour cells appear to do this by hijacking caspase signalling in order to limit the innate sensibility of mtDNA in the cytoplasm and further limit anti‐tumour immunity in the tumour microenvironment. So it is clear that miMOMP promotion can enhance the therapeutic efficacy of multiple anti‐tumour therapies and provide assistance in reducing the intrinsic drug resistance of tumour cells. In general, the risk of developing tumours increases with age. When we develop drugs targeting miMOMP inhibition to counteract SASP to ameliorate cellular senescence or to treat age‐associated diseases, we take into account that mtDNA in the cytoplasm exerts a positive gain effect on tumour therapy when miMOMP is promoted, and that this therapy may put concurrent anti‐tumour therapies at risk of failing or causing unpredictable consequences for the target patient if he or she suffers from certain tumour diseases. One potential therapeutic strategy we can adopt is to develop different subtypes of miMOMP inhibitors. When tumour patients are treated with miMOMP inhibitory drugs, specific miMOMP inhibitory drug subtypes should be used. This subtype can be employed to specifically exclude the location of the tumour based on utilising specific information, such as the location and nature of the tumour. This allows the treatment to reach the ageing parts of the body, such as the brain, bones and heart to exert a therapeutic effect. In the event that the tumour is of a multi‐focal or metastatic nature, and if it is situated within a region associated with age‐related degeneration, alternative chemotherapy regimens that are less likely to develop drug resistance and immune evasion may be employed concurrently. The aforementioned approach represents a potential experimental design worthy of further consideration as part of a programme of research and development in the future.

In conclusion, we must be very cautious about applying inhibition of miMOMP to anti‐SASP tumour patients. Before the final clinical application, some drug trials and in‐depth studies in oncology patients are still needed to control the risk and evaluate the efficacy. Moreover, these drugs have potential off‐target effects, and risk control and rational experimental design are more important. The above will provide new ideas for drug regulation of SASP, intervention in the treatment of age‐related diseases and human anti‐ageing.

## AUTHOR CONTRIBUTIONS

Yang Xuan wrote the manuscript. Yang Xuan and Yue Duan revised the manuscript. All authors contributed to the article and approved the submitted version.

## FUNDING INFORMATION

This work and related studies were supported by the Natural Science Foundation of Zhejiang Province (grant No.Y20H270024) and Zhejiang Traditional Chinese Medicine Administration (grant No. 2022ZZ017).

## CONFLICT OF INTEREST STATEMENT

None declared.

## Data Availability

Data sharing is not applicable to this article as no new data were created or analyzed in this study.
